# Newly Identified Electrically Coupled Neurons Support Development of the *Drosophila* Giant Fiber Model Circuit

**DOI:** 10.1523/ENEURO.0346-18.2018

**Published:** 2018-12-14

**Authors:** Tyler Kennedy, Kendal Broadie

**Affiliations:** 1 Department of Biological Sciences, Vanderbilt University and Medical Center, Nashville, Tennessee 37235; 2 Department of Cell and Developmental Biology, Vanderbilt University and Medical Center, Nashville, Tennessee 37235; 3 Vanderbilt Brain Institute, Vanderbilt University and Medical Center, Nashville, Tennessee 37235

**Keywords:** circuit map, electrical synapse, innexin

## Abstract

The *Drosophila* giant fiber (GF) escape circuit is an extensively studied model for neuron connectivity and function. Researchers have long taken advantage of the simple linear neuronal pathway, which begins at peripheral sensory modalities, travels through the central GF interneuron (GFI) to motor neurons, and terminates on wing/leg muscles. This circuit is more complex than it seems, however, as there exists a complex web of coupled neurons connected to the GFI that widely innervates the thoracic ganglion. Here, we define four new neuron clusters dye coupled to the central GFI, which we name GF coupled (GFC) 1–4. We identify new transgenic Gal4 drivers that express specifically in these neurons, and map both neuronal architecture and synaptic polarity. GFC1–4 share a central site of GFI connectivity, the inframedial bridge, where the neurons each form electrical synapses. Targeted apoptotic ablation of GFC1 reveals a key role for the proper development of the GF circuit, including the maintenance of GFI connectivity with upstream and downstream synaptic partners. GFC1 ablation frequently results in the loss of one GFI, which is always compensated for by contralateral innervation from a branch of the persisting GFI axon. Overall, this work reveals extensively coupled interconnectivity within the GF circuit, and the requirement of coupled neurons for circuit development. Identification of this large population of electrically coupled neurons in this classic model, and the ability to genetically manipulate these electrically synapsed neurons, expands the GF system capabilities for the nuanced, sophisticated circuit dissection necessary for deeper investigations into brain formation.

## Significance Statement

Genetic model neural circuits with individually identifiable neurons help us to understand how nervous systems wire together during development, and then operate through coordinated chemical and electrical signaling. The *Drosophila* giant fiber circuit has long served as such a model, due to large neuron size, genetic malleability, and easily visualized behavioral output: a jump in response to a threat. This study unveils new members of this circuit, all of which synapse with the circuit at one site on the central giant fiber interneuron. We use new tools to identify and transgenically manipulate these neurons and show that these neurons are required for proper circuit development. This study provides a detailed circuit map for further dissection of neuronal connectivity and electrically coupled communication.

## Introduction

The *Drosophila* giant fiber (GF) circuit is particularly suitable for single-neuron resolution neurodevelopmental studies for a number of reasons, all related to its role as an escape response circuit (Allen et al., 2006; Boerner and Godenschwege, 2011). The need for rapid signal conduction from the senses through brain to muscles promoted the evolution of very large neurons throughout this circuit, facilitating their visualization and manipulation (Power, 1948; Borgen et al., 2017). This enlargement is most prominent in the long-distance GF interneuron (GFI), which consolidates sensory information in the brain and projects through the neck into the thoracic ganglion (TG) via giant axons (Allen et al., 1998; Pézier et al., 2014). To increase communication speed and fidelity between neurons, the GF circuitry uses mixed chemical and electrical synapses (Thomas and Wyman, 1984; Blagburn et al., 1999; Fayyazuddin et al., 2006). These electrical synapses, composed of the Shaking-B innexin, can pass small tracer dyes to identify coupled partner neurons (Phelan et al., 1996).

The GF circuit targets two large muscle sets used for rapid escape behavior, the tergotrochanteral muscle (TTM), which controls the legs for jumping, and the dorsal longitudinal muscle (DLM), which controls the wings (Tanouye and Wyman, 1980). The escape behavior is easily scored, and muscles are accessible to electrophysiological recordings, providing two outlets to study whole-circuit function (Martinez et al., 2007; Augustin et al., 2011; von Reyn et al., 2014). The GFI connects to the TTM via the tergotrochanteral motoneuron (TTMn) and to the DLM via the peripherally synapsing interneuron (PSI), which in turn synapses onto the dorsal longitudinal motoneuron (Tanouye and Wyman, 1980; Allen et al., 2006). While the GF circuit is reported to be quite simple, electrophoretic injections with small dyes make it clear that the GFI is actually part of a much larger circuit network of undescribed neurons (Boerner and Godenschwege, 2011; Enneking et al., 2013; Kennedy and Broadie, 2017).

This larger GF circuit should come as no surprise, as most classically studied circuits are continuously being updated to include new neurons, increasing the appreciation of the complexity and interconnectivity within the brain (Lin et al., 2016; Talay et al., 2017; Cande et al., 2018). Describing the wiring diagrams of classic circuits within model brains is important for understanding how local circuits accomplish processing tasks while also overriding or promoting behaviors controlled by separated but interconnected circuits (Gaudry and Kristan, 2009; Stensmyr et al., 2012; von Reyn et al., 2014). More complex model circuits can better help to answer questions about how circuits develop and evolve over time (Ward et al., 2015; Tosches, 2017). Combining GF circuit manipulability with the full complement of GFI-coupled neurons should enable robust new avenues for experimentation on how neurons select partners, determine synaptic strength, and regulate neighboring circuits.

In this study, we use neurobiotin (NB) dye injection to map previously uncharacterized GF-coupled (GFC) neurons. We take advantage of the FlyLight Gal4 library collection to identify transgenic drivers for the GFC neurons (Brand and Perrimon, 1993; Jenett et al., 2012). This approach defined four new GFI-coupled neuron clusters (i.e., GFC1–4) within the GF circuit, which we characterize for their architecture, neuronal polarity, and synaptic connectivity. We show that the inframedial bridge (IB; Allen et al., 1998) is the GFI site where all the GFC neurons come together to synapse with the circuit. We ablate GF neurons by transgenic expression of the apoptotic head involution defective (Hid) protein (Zhou et al., 1997) to find that GFC1 and PSI are required for proper GFI development. We also find GFI axons always compensate for the loss of their bilaterally symmetric partner through new contralateral innervation. Together, this work broadens the known GF circuit and opens new avenues for studying electrically coupled circuit development, function, and plasticity.

## Materials and Methods

### *Drosophila* genetics

All animals were maintained on a standard cornmeal/agar/molasses *Drosophila* food diet in a 12 h light/dark cycling incubator at 25°C. Timed-lay eggs were collected for 2–3 d, and experimental animals were selected from rearing tubes 10–14 d later. The following *Drosophila* lines were used for genetic crosses: *w^1118^* (RRID:BDSC_3605); *w^1118^;* P{GMR78A06-GAL4}attP2 (Jenett et al., 2012; RRID:BDSC_46999); *w^1118^;* P{GMR73C07-GAL4}attP2 (RRID:BDSC_46689); *w^1118^;* P{GMR24H07-GAL4}attP2 (RRID:BDSC_49317); *w^1118^;* P{GMR42A06-GAL4}attP2 (RRID:BDSC_41245); *w^1118^;* R10B11-p65.AD}attP40 (Dionne et al., 2018; RRID:BDSC_68807); *w^1118^;* P{GMR14A06-GAL4.DBD}attP2 (RRID:BDSC_68738); *w^1118^*, *y^1^*; 10X UAS-*ivs*-*mCD8*::*GFP* attP40 (Pfeiffer et al., 2010); UAS-*hid*.Z/CyO (Zhou et al., 1997; RRID:BDSC_65403); and *w^1118^;* UAS-*DenMark*, UAS-*syt::GFP* (Zhang et al., 2002; Nicolai et al., 2010; RRID:BDSC_33064). Both females and males were used in this study, with sex-specific selection stated in figure legends. All genotypes were verified with visible markers.

### Dye iontophoresis

GFI dye injections were performed similar to the previously published methods (Boerner and Godenschwege, 2011; Kennedy and Broadie, 2017). Briefly; glass electrodes (Kwik-Fil Borosilicate Glass 1B100F-4, World Precision Instruments) were pulled on a laser electrode puller (Model P-2000, Sutter Instrument) to 10 MΩ resistance (3 m KCl). Electrodes were filled with 0.25% tetramethylrhodamine isothiocyanate (TRITC)-dextran (10 kDa; Life Technologies) and 7% neurobiotin (Vector Laboratories; RRID:AB_2313575) in double-distilled dH_2_O. Filled electrodes were placed on a silver chloride wire mounted on a PCS-5000 Micromanipulator (Burleigh). Animals in physiologic saline were cut along the dorsal midline to access the cervical connective (CC), at which electrodes were inserted into the GFI. A square-pulse stimulator (Grass S48, Astro-Med) provided 7.5 100 ms pulses/s for 2 min with the 20 nA injected current monitored by an AxoClamp2B Amplifier. A Digidata data acquisition system (1320A, Molecular Devices) was controlled with Clampex 9.2 software.

### Confocal imaging

Brains were fixed for 30 min in 4% paraformaldehyde/sucrose (Electron Microscopy Services) in PBS (pH 7.2; Life Technologies) and then rinsed 3× with PBS, and blocked for 1 h with 1% bovine serum albumin (BSA; Sigma-Aldrich) in PBST (PBS + 0.2% Triton X-100; Thermo Fisher Scientific). Labels were diluted in PBST with 0.2% BSA. The following labels were used: streptavidin::Cy5 (1:20; Life Technologies); rabbit anti-ShakB (1:250; Phelan et al., 1996); rabbit anti-GFP (1:2000; Abcam; RRID:AB_303395); FITC goat anti-GFP (1:500; Abcam; RRID:AB_305635); rabbit anti-RFP (1:500; Rockland; RRID:AB_2209751); Alexa Fluor 488-conjugated donkey anti-goat (1:250; Thermo Fisher Scientific; RRID:AB_2534102); Alexa Fluor 488-conjugated donkey anti-rabbit (1:250; Thermo Fisher Scientific; RRID:AB_2556546); Alexa Fluor-568-conjugated donkey anti-rabbit (1:250; Thermo Fisher Scientific; RRID:AB_2534017); Alexa Fluor-647 conjugated donkey anti-rabbit (1:250; Thermo Fisher Scientific; RRID:AB_2536183); and Alexa Fluor-633-conjugated goat anti-rabbit (1:250; Thermo Fisher Scientific; RRID:AB_141419). Next, preparations were rinsed 3× for 30 min in PBST and 1× in PBS, and then were mounted on glass microscope slides (Probe On Plus 25 × 75 × 1.0 mm, Thermo Fisher Scientific) in 2,2´-thiodiethanol (Sigma-Aldrich; Staudt et al., 2007). To prevent crushing, double-sided poster tape (Scotch) was placed on each side of the brains. Coverslips (catalog #1.5H, Zeiss) were sealed with nail polish (Hard as Nails, Sally Hansen). Fluorescent images were collected using a Zeiss LSM 880 Confocal Microscope with an AiryScan module, which has increased lateral resolution (161 nm) and signal-to-noise ratio (Sivaguru et al., 2016). Images show maximum *Z*-stack projections under standard confocal mode, unless otherwise noted in the figure legends.

### Data analyses

Data processing and image creation were performed with FIJI software (version 2; RRID:SCR_002285; Schindelin et al., 2012; Schneider et al., 2012). Neuronal models and movies were created using Imaris (version 9.2; RRID:SCR_007370).

## Results

### The giant fiber circuit exhibits extensive dye-coupled connectivity

**Figure 1.**
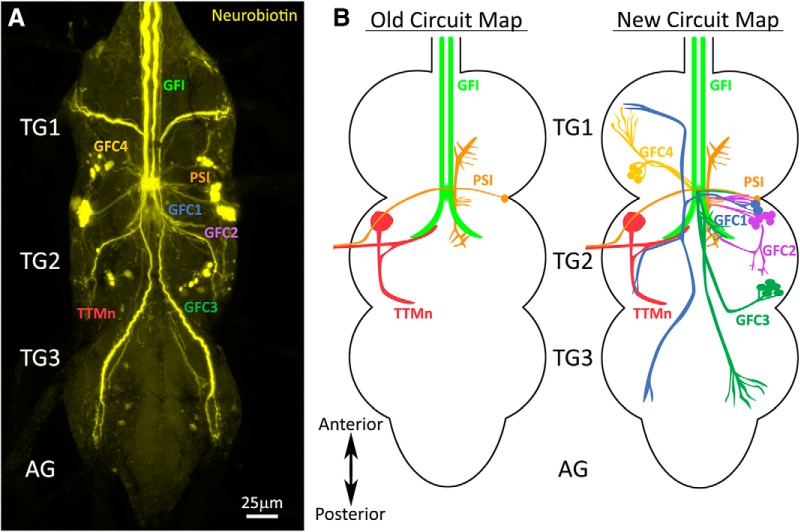
Giant fiber interneuron dye injection reveals coupled neurons. ***A***, The GFI iontophoretically injected with neurobiotin (yellow) shows extensive dye coupling to neurons in the TG. The established GFI-coupled neurons are (1) the PSI (orange) and (2) the TTMn (red). The newly identified GFCs project into all three TG segments (TG1–3), but do not extend into the AGs. ***B***, Left, The old GF circuit map showing both of the previously characterized GFI (green) dye-coupled neurons: PSI (orange) and TTMn (red). Right, The new GF circuit map with the addition of all the newly identified GFC neurons from this study: GFC1 (blue), GFC2 (purple), GFC3 (dark green), and GFC4 (yellow).

Small gap junction-permeable dyes used to study the GF circuit have consistently revealed an extensive, but uncharacterized, network of dye-coupled neurons (Boerner and Godenschwege, 2011; Enneking et al., 2013; Kennedy and Broadie, 2017). To thoroughly study the architecture and properties of these neurons, we iontophoretically injected the GFI with the highly gap junction-permeable NB dye, and then labeled the brains *post hoc* with a streptavidin-conjugated fluorophore (Huang et al., 1992). Consistent with previously published work, this intracellular dye injection reveals an extensive network of neurons dye coupled to the GFI (Fig. 1). This dye coupling is the direct result of gap junction connectivity, as eliminating gap junctions using *shaking-B* mutants (*shakB^2^*) prevents all NB dye transfer (data not shown; Blagburn et al., 1999; Kennedy and Broadie, 2017). A summary of this newly identified GF circuitry is shown in Figure 1.

Although there are a large number of dye-labeled processes widely distributed throughout the TG (Fig. 1*A*), all published GF circuit maps name only two GFI-coupled cells: (1) TTMn and (2) PSI (Fig. 1*B*, old circuit map). Here, we map and characterize all of the dye-coupled neurons whose projections we can trace back to an identifiable cell soma. We have named these neurons GFC followed by an identifying number (Fig. 1*A*,*B*). In this study, we report the characterization of four neuron clusters (GFC1–4), each of which represents a bilaterally symmetric set of two to seven neurons (Fig. 1*B*, new circuit map). The processes of these neurons contact the descending GFI axons and reach into all three TG segments (TG1–3), but do not cross into the brain or abdominal ganglion (AG). To understand how the GFCs integrate into the GF circuit, we began by obtaining selective genetic access to these neurons.

### Transgenic Gal4 drivers for newly identified giant fiber-coupled neurons

**Figure 2.**
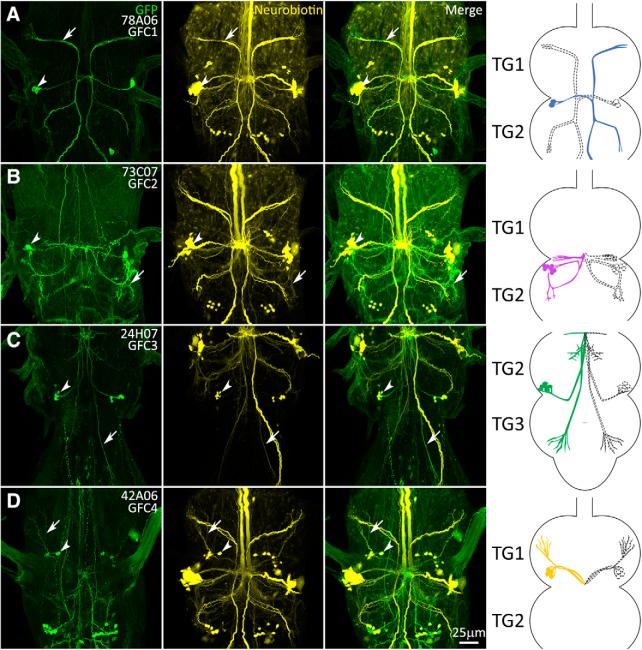
Transgenic Gal4 drivers for the newly identified GFC neurons. Gal4-driven expression of UAS-*mCD8::GFP* (green, column 1) overlapping with the GFI injection of neurobiotin dye (yellow, column 2) showing the identification of GFC drivers (merge, column 3). Arrows indicate processes with overlapping GFP and NB labeling, and arrowheads show the GFC cell bodies. The GFC neurons are drawn both in color (Fig. 1 color scheme) and perforated outlines to show their bilateral pattern (column 4). TG segments are selected to best show GFC projection architecture. All injections were performed on females. ***A***, 78A06-Gal4 labels GFC1. The driver strength is relatively weak, with a somewhat stochastic labeling of the GFC1 neurons. ***B***, 73C07-Gal4 labels GFC2. This driver is moderately strong, but also labels other neurons. ***C***, 24H07-Gal4 labels GFC3. This driver strength is moderate, with labeling of other neurons. ***D***, 42A06-GAL4 labels both GFC3 and GFC4 neurons. The driver is relatively weak, with stochastic labeling of GFC4 neurons.

To accurately map and manipulate the separate GFC neuron populations, we set forth to identify Gal4 drivers with highly specific expression for each GFC using two approaches. First, we conducted an *in silico* screen through the entire Janelia FlyLight library, which includes lines generated from the Vienna Tiles project (9436 lines; Jenett et al., 2012; Tirian and Dickson, 2017). Using images of the GFI dye-labeled circuit (Fig. 1*A*), we screened for matching GFP expression patterns (Fig. 2). We identified highly specific Gal4 drivers for GFC1 (78A06; Fig. 2*A*) and GFC2 (73C07; Fig. 2*B*), as well as less specific drivers for GFC3 and GFC4. Second, for cleaner GFC3 and GFC4 drivers, we used the recent automated Color-Depth Maximum Intensity Projection (MIP) tool for the *Drosophila* transgenic database (Otsuna et al., 2018). Using the less specific driver lines as templates to search this library, we screened for specific Gal4 drivers for GFC3 and GFC4. This complementary approach uncovered a highly specific driver for GFC3 (24H07; Fig. 2*C*) and a combined driver for GFC3/4 (42A06; Fig. 2*D*). During our search with the MIP tool, we identified many additional GF circuit drivers, aside from the ones used in this study. We selected the cleanest drivers and report them in Table 1 for use in future experiments.

**Table 1:** Transgenic Gal4 driver lines for the giant fiber circuit

GFI	GCI	TTMn	PSI	GFC1	GFC2	GFC3
R14A01	R32C04	R25D08	R26E04	R93E07	R13C08	R44D02
VT004455	R74E09	R88F07	R75E05	R87D02	R77C12	R58E04
VT042336	VT002209	VT038335	VT030598	VT059438	VT043662	R75D03

New Gal4 drivers (distinct from those used in this study) that express selectively within the GF circuit, as compiled from the Janelia FlyLight and Vienna Tiles library collections. Selective lines for GFC4 have not been uncovered and thus are not reported here.

To confirm that the new Gal4 transgenic driver lines label the bona fide GFC components of the GF circuit, we crossed each Gal4 line with the UAS-*mCD8::GFP* membrane reporter (Fig. 2, column 1) and injected the GFI with NB (Fig. 2, column 2). The merged images show perfect overlap between each transgenic driver line and the specified subset of the dye-labeled neurons (Fig. 2, column 3). Cell bodies are strongly labeled in all cases (Fig. 2, arrowheads), and individual neuronal processes of GFC1–4 can be traced for both the GFP and NB signals (Fig. 2, arrows). However, in some cases, such as GFC2 (73C07-Gal4), the dye injection signal is much dimmer than for other neurons, such as GFC1 (78A06-Gal4). Each GFC cluster is schematically represented within the TG, with full color on one side (Fig. 1, color scheme) and dashed outlines on the other side, to show each individual GFC neuron as well as their bilaterally symmetrical pattern (Fig. 2, column 4). Using these Gal4-driven GFP expression patterns, we are able to map each GFC cluster within the TG.

### Projection architecture of GFC neurons within the thoracic ganglion

GFC1 is composed of two bilaterally symmetrical neurons on each side of TG2 (Fig. 2*A*). Each soma projects a process medially, which crosses the midline at the IB (Allen et al., 1998) and then splits, sending one branch anteriorly and one posteriorly. The anterior process travels halfway up TG1, then bends laterally and ventrally to terminate in the anterior corner of TG1, almost at the ventral-most point of the TG (Fig. 2*A*). This process extends several thin terminals, beginning in the same plane as the GFI bend. The posterior process splits halfway down TG2, just below the GFI bend. One branch proceeds laterally, then turns posteriorly toward the TG2 edge, with a ventral dive and several thin terminals, before terminating in the TG2 posterior lateral corner (Fig. 2*A*). The other process descends into TG3, bends inward toward the midline, then laterally to the anterior edge. From here, the process projects posteriorly and ventrally to end in a fashion similar to that of the other two terminals (Fig. 2*A*). All three GFC1 projections appear to innervate the leg neuropils (Namiki et al., 2018).

The seven bilaterally symmetric GFC2 neurons are largely restricted to TG2 (Fig. 2*B*). These cell bodies neighbor GFC1 and similarly project fasciculating processes medially. However, two-thirds of the way to the midline, the processes bend posteriorly and then laterally, to curve ventrally toward the lower edge of TG2 in the region of the GFI axon bend (Fig. 2*B*). The processes then curve anteriorly back toward the cell bodies, with a slight dorsal trajectory before termination, projecting several short, heavily branched termini in anterior and posterior directions. Another process doubles back toward the posterior deflection, travels medially to the midline and then sends out two branches posteriorly (Fig. 2*B*). One curves ventrolaterally to terminate along the first ventral spiral, and the other travels dorsolaterally along the path of the original anterior process, terminating as it turns up toward the soma. There are two other processes that depart from the midline: one travels dorsally and slightly posteriorly before terminating, and one projects anteriorly and dorsolaterally to terminate in the lower central TG1 (Fig. 2*B*). These processes both appear to innervate the wing neuropils (Namiki et al., 2018).

GFC3 is composed of five bilaterally symmetrical neurons with the cell bodies positioned dorsally in the posterolateral corner of TG2 (Fig. 2*C*). These cells send out fasciculating processes that first proceed ventrally in a medial–anterior direction up to the central IB connection with the GFI. At the IB, extensive GFC3 branches are visible, extending laterally and dorsally, but no further in either the anterior or ventral direction (Fig. 2*C*). These processes also track along the large terminal bend of the GFI axon. Dorsal to the IB, the main GFC3 processes reverse course to travel posterolaterally, while remaining ipsilateral to their cell bodies. The projection direction is ventral until TG3 is reached, at which point the processes move dorsally once again (Fig. 2*C*). These processes terminate near the anterior portion of TG3 within the leg neuropil, in a series of thin processes at approximately the same axial plane as the IB and GFI axonal bends (Fig. 2*C*). Of note, both GFC1 and GFC3 were unintentionally captured in a recent screen for descending neurons (Namiki et al., 2018).

The four bilaterally symmetric GFC4 neurons are largely restricted to TG1 (Fig. 2*D*). The GFC4 cell bodies lie in the TG1 dorsal lateroposterior corner. The GFC4 processes first fasciculate to project ventrally, then posteromedially, running to the central IB (Fig. 2*D*). From the IB, the GFC4 processes then reverse course, remaining ipsilateral to their cell bodies as they project dorsally, back the way they came toward their cell bodies (Fig. 2*D*). When the GFC4 processes are directly below their cell bodies, they turn ventrally, and then travel toward the TG1 anterolateral corner to terminate in long finger-like projections (Fig. 2*D*). Like the other GFCs, the GFC4 processes appear to innervate the leg neuropils (Namiki et al., 2018). Overall, these transgenic driver lines allow detailed analysis of GFC architecture, and provide highly specific genetic control over the GFC neurons. To determine how these neurons interact with the GF circuit, we next examined their contact points with the GFI.

### The inframedial bridge connectivity site of GFI–GFC intersection

**Figure 3.**
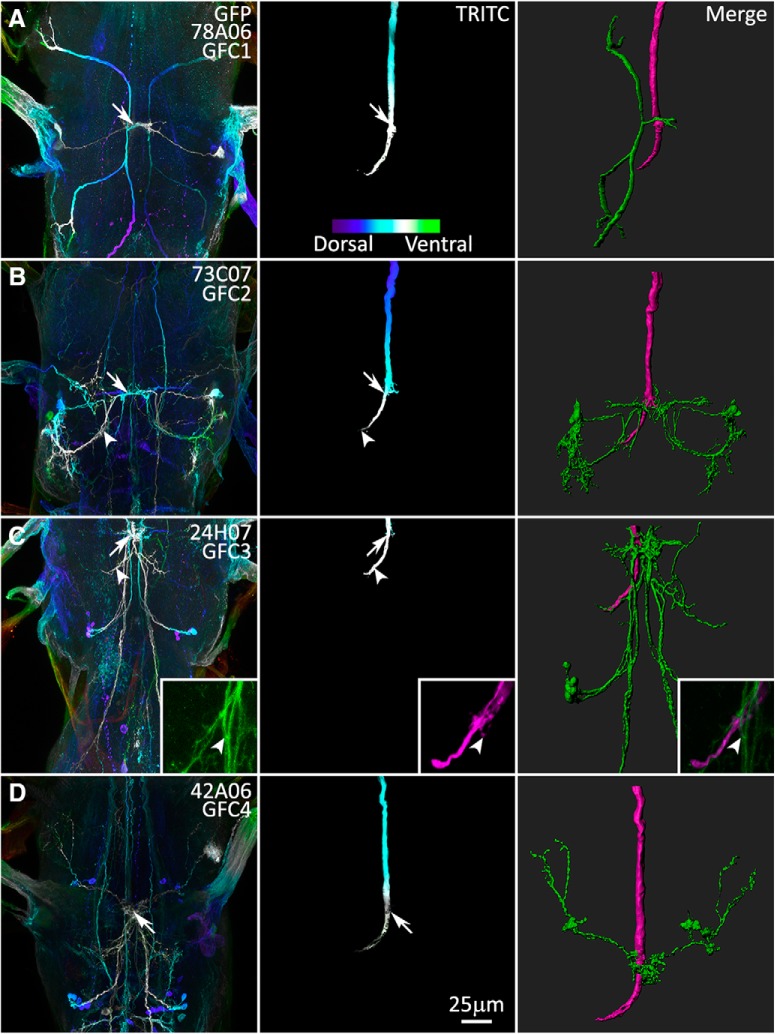
The GFI interacts with the GFC neurons at the inframedial bridge. Gal4 lines driving UAS-*mCD8::GFP* (column 1) intersect with the GFI axon revealed by injection of TRITC (column 2), at the GFI IB and the GFI axonal bend (merge, column 3). The first two columns use depth color coding to represent the *Z*-position within the TG, with more dorsal regions displaying cool colors and ventral regions displaying warm colors (see color scale bar in ***A***, column 2). Arrows indicate overlapping membrane contact between GFCs and GFI at the IB. Arrowheads indicate GFC contact at the GFI axon bend. All injected flies are female. ***A***, GFC1 (78A06-Gal4) interacts with the GFI exclusively at the IB. ***B***, GFC2 (73C07-Gal4) interacts with the GFI at the IB and the GFI axonal bend. ***C***, GFC3 (24H07-Gal4) interacts with the GFI extensively at the IB and the GFI axonal bend. The GFI also produces small side projections that contact GFC3 (inset, arrowheads). ***D***, GFC4 (42A06-Gal4) interacts with the GFI at the IB.

GFC1–4 are all dye coupled to GFI via direct or indirect gap junction connections (Fig. 1), and all of these neurons project to the central IB to overlap with the GFI (Fig. 2). The IB has been defined as a region proximal to the GFI lateral axonal bend, where the GFI axon puts forth tufted projections and connects to the PSI (Allen et al., 1998). We therefore hypothesized that the IB is the primary site of GFI–GFC connectivity. To determine the location of potential synaptic sites between the GFI and GFCs, we injected the GFI with the large, nonpermeant dye TRITC-dextran (10 kDa; Boerner and Godenschwege, 2011; Enneking et al., 2013; Kennedy and Broadie, 2017) for all the UAS-*mCD8::GFP*-labeled GFC1–4 lines (Fig. 3). We then assayed for overlap regions where the GFC membrane signal (Fig. 3, column 1) contacts the GFI TRITC signal (Fig. 3, column 2). Merging the two channels to create static (Fig. 3, column 3) and dynamic (Movies 1-4) 3D reconstructions of the spatial overlap provides clear identification of GFI–GFC contact points.

GFP and TRITC signals are color coded by depth to visualize the *Z* dimension (FIJI plugin: Temporal-Color Code), with neurons proximal in *Z* space displaying the same color (Fig. 3, columns 1 and 2). Overlap between neurons is shown for GFC neurons (Fig. 3, green) and GFI (Fig. 3, column 3, magenta). The results show that GFC1 contacts the GFI only at the IB with a simple crossing branch (Fig. 3*A*, arrows, Movie 1). GFC2–4 also contact the GFI directly at the IB, but with a much higher level of complexity (Fig. 3*B–D*, arrows, Movies 2-4). Further, GFC2 and GFC3 have processes that branch from the IB and overlap the large terminal bend of the GFI axon (Fig. 3*B*,*C*; arrowheads, Movies 2, 3). This is the first example, to our knowledge, of any contact along the GFI axonal bend other than TTMn. We also observe a third contact point between GFI and GFC3. The GFI axon bend occasionally extends small processes, which can contact GFC3 on posteriorly descending processes (Fig. 3*C*, arrowhead; magnified in inset). As these overlaps are likely sites for gap junction connectivity within the circuit, we investigated these membrane contacts for electrical synapses.

Movie 1. 3D animation of GFC1 and GFI interaction. Animated 3D reconstruction of mCD8::GFP-labeled GFC1 (green) and TRITC-injected GFI (magenta) in thoracic ganglion segments 1 and 2 (TG1/2). GFC1 intersects with the GFI in a narrow projection that crosses the IB. This projection then splits to create claw-like synaptic terminals in TG1–3 (TG3 not pictured). Scale bar, 20 μm. 10.1523/ENEURO.0346-18.2018.video.1

Movie 2. 3D animation of GFC2 and GFI interaction. Animated 3D reconstruction of mCD8::GFP-labeled GFC2 (green) and TRITC-injected GFI (magenta) in TG1/2. GFC2 extends a large TG2 loop with dorsal projections. GFC2 intersects with the GFI extensively at the IB and to a lesser extent at the tip of the TG2 axonal bend. Scale bar, 20 μm. 10.1523/ENEURO.0346-18.2018.video.2

Movie 3. 3D animation of GFC3 and GFI interaction. Animated 3D reconstruction of mCD8::GFP-labeled GFC3 (green) and TRITC-injected GFI (magenta) in TG2/3. GFC3 cell bodies project processes to the IB and contact the GFI, with extensive branching, including along the GFI axonal bends. GFC3 then projects into TG3 to terminate. Scale bar, 20 μm. 10.1523/ENEURO.0346-18.2018.video.3

Movie 4. 3D animation of GFC4 and GFI interaction. Animated 3D reconstruction of mCD8::GFP-labeled GFC4 (green) and TRITC-injected GFI (magenta) in TG1/2. GFC4 cell bodies project processes from TG1 to the IB, then reverse course and return to TG1 where they terminate. Scale bar, 20 μm. 10.1523/ENEURO.0346-18.2018.video.4

### Shaking-B gap junction synapses between GFI and GFC neurons

**Figure 4.**
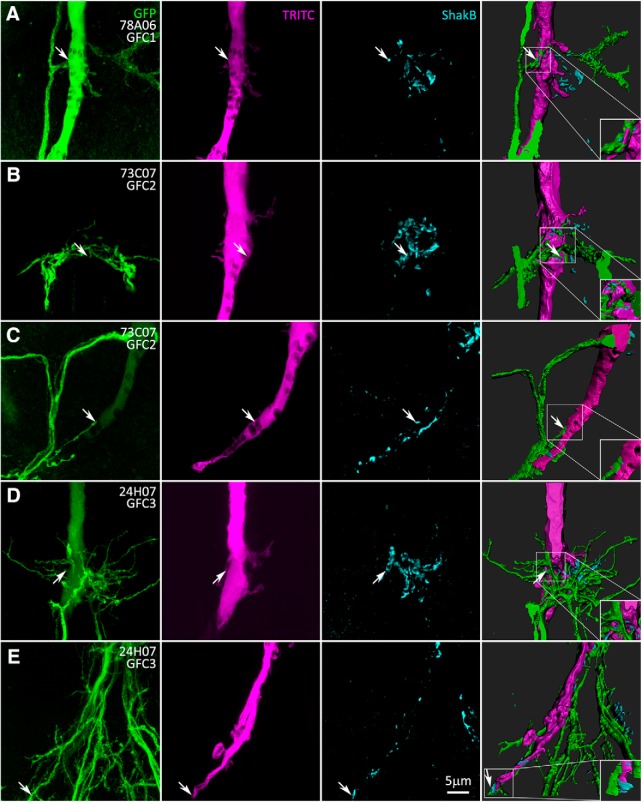
GFCs form electrical synapses with the GFI at the inframedial bridge. Electrical synapses between GFI and GFC neurons are shown in Gal4-driven UAS-*mCD8::GFP* animals (green, column 1) with TRITC dye injection into the GFI (magenta, column 2), while colabeling with the Shaking-B antibody (cyan, column 3). Images were taken using the AiryScan mode of the microscope. The three merged channels (column 4) show the regions of shared ShakB contact between GFI-GFCs. Arrows indicate sites of the GFI–GFC ShakB synaptic contacts (magnified in insets). All injected flies are female. ***A***, GFC1 (78A06-Gal4) makes ShakB electrical synapse contacts with the GFI at the IB. ***B***, GFC2 (73C07-Gal4) forms several ShakB electrical synapse contacts with the GFI. ***C***, GFC2 (73C07-Gal4) contacts the GFI along the axonal bend. ***D***, GFC3 (24H07-Gal4) contacts the GFI with multiple ShakB electrical synapses. ***E***, GFC3 (24H07-Gal4) minimally contacts the GFI along the axonal bend (arrow).

The GF circuit is characterized by mixed chemical and electrical synapses (Blagburn et al., 1999; Allen et al., 2006). To map GFI–GFC electrical synapses, we labeled for the Shaking-B (ShakB) innexin, using an antibody recognizing the “*N* + 16” isoform present at GFI synapses (Phelan et al., 2008). Flies in which GFC1–4 neurons are labeled with UAS-*mCD8::GFP* (Fig. 4, column 1, green) were GFI injected with TRITC (Fig. 4, column 2, magenta) and colabeled with ShakB antibody (Fig. 4, column 3, cyan). All three channels were modeled with 3D rendering software to visualize ShakB-positive GFI–GFC contacts (Fig. 4, column 4, Movies 5–9). GFC1 (78A06-Gal4) exhibits a simple arborization, with a process coming across the IB, and making a characteristic anterior–posterior split (Fig. 4*A*, Movie 5). ShakB is clearly visible in the 3D models, localized between the GFI and GFC1 as the process exits the IB (Fig. 4, arrows and inset). GFC1 projects axons to all three TG segments, indicating that there is a set of outputs triggered by the GFI escape response in parallel to TTM and DLM activation.

Movie 5. 3D animation of ShakB electrical synapses between GFC1 and GFI at IB. Animated 3D reconstruction of mCD8::GFP-labeled GFC1 (green), TRITC-injected GFI (magenta), and anti-ShakB electrical synapse labeling (cyan). The simple passing dendrite of GFC1 interacts with the GFI at multiple locations within the IB. Multiple sites of ShakB electrical synapses indicate direct GFC1–GFI coupling. Scale bar, 5 μm. 10.1523/ENEURO.0346-18.2018.video.5

Movie 6. 3D animation of ShakB synapses between GFC3 and GFI at the axonal bend. Animated 3D reconstruction of mCD8::GFP-labeled GFC3 (green), TRITC-injected GFI (magenta), and anti-ShakB electrical synapse labeling (cyan). GFC3 extensively contacts the GFI along the GFI axonal bends in TG2. Despite this extensive contact, there are minimal ShakB punctae (cyan) shared between the neurons. Scale bar, 5 μm. 10.1523/ENEURO.0346-18.2018.video.6

GFC2 neurons have a larger process field, forming a hemicircle in front of the GFI (Fig. 4*B*, Movie 6). Multiple ShakB electrical synapses clearly occur between the GFI and GFC2, although, due to the complexity of these connections, it is not possible to determine whether the GFI is contacting the GFC2 processes that come from the contralateral or ipsilateral sides of the TG, or both (Fig. 4*B*). GFC2 also contacts the GFI along the distal axonal bend (Fig. 3*B*), so we also investigated these sites for ShakB colocalization. The results show contact between the GFI and GFC2 near the tip of the bend; however, ShakB punctae are rarely seen colocalizing at these contacts (Fig. 4*C*, Movie 7), suggesting that these are primarily chemical synapse connections.

Movie 7. 3D animation of ShakB synapses between GFC2 and GFI at IB. Animated 3D reconstruction of mCD8::GFP-labeled GFC2 (green), TRITC-injected GFI (magenta), and anti-ShakB electrical synapse labeling (cyan). The GFC2 field interacts in multiple locations with the GFI, including several side projections from the IB. Several sites of ShakB electrical synapses indicate GFC2–GFI coupling. Scale bar, 5 μm. 10.1523/ENEURO.0346-18.2018.video.7

Movie 8. 3D animation of ShakB synapses between GFC2 and GFI at axonal bend. Animated 3D reconstruction of mCD8::GFP-labeled GFC2 (green), TRITC-injected GFI (magenta), and anti-ShakB electrical synapse labeling (cyan). GFC2 contacts the GFI along the TG2 axonal bends, mostly at the tips. Along these contact sites, there are few to no ShakB contacts (cyan) shared between the neurons. Scale bar, 5 μm. 10.1523/ENEURO.0346-18.2018.video.8

GFC3 has the most extensive IB contacts among all the GFCs, as well as broad interactions with surrounding neurons (Fig. 4*D*, Movie 8). GFC3 contacts the GFI with ShakB electrical synapses (Fig. 4*D*, arrows), but GFC3 branches extending beyond the IB are mostly ShakB negative (Fig. 4*D*), indicating few electrical synapses. GFC3 contacts the GFI axon bend even more extensively than GFC2, but similarly has a small number of ShakB electrical synapse contacts (Fig. 4*E*, Movie 9). All images of GFI–GFC3 IB contact sites exhibit ShakB-positive electrical synapses, but only one image of the GFI–GFC3 axonal bend shows a synaptic connection (Fig. 4*E*, arrow). GFI axon bends are presynaptic to the TTMn, with extensive ShakB electrical synapses (Phelan et al., 2008), but it appears that only a small portion of this gap junction connectivity is used for GFC2 and GFC3, with the primary GFI–GFC electrical connections in the IB (Fig. 4*B*,*D*). Without a GFC4-specific driver, we are unable to specifically test GFI–GFC4 ShakB synaptic connections. To determine the direction of information flow across GFI–GFC synapses, as well as connectivity in other regions of the TG, we next mapped the presynaptic and postsynaptic neuronal polarity of GFC1–4 synapses.

Movie 9. 3D animation of ShakB synapses between GFC3 and GFI at IB. Animated 3D reconstruction of mCD8::GFP-labeled GFC3 (green), TRITC-injected GFI (magenta), and anti-ShakB electrical synapse labeling (cyan). GFC3 extends the largest dendritic field at the IB, with extensive GFC3–GFI contact. Several of these contact points are positive for ShakB electrical synapses. Scale bar, 5 μm. 10.1523/ENEURO.0346-18.2018.video.9

### Presynaptic and postsynaptic polarity of thoracic ganglion GFC neurons

**Figure 5.**
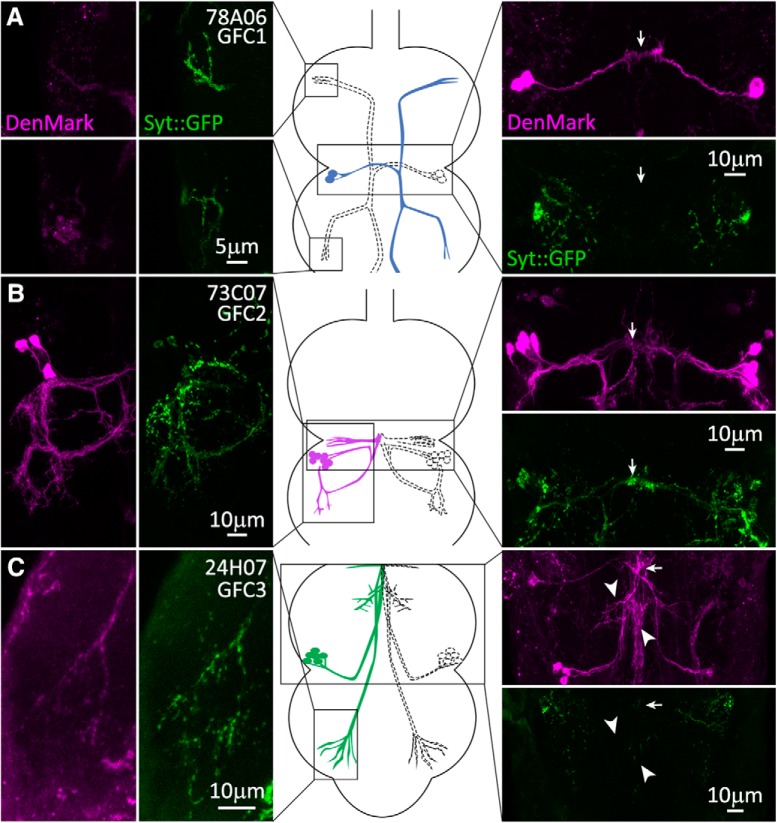
Presynaptic and postsynaptic polarity of the newly identified GFC neurons. GFC neuronal polarity is shown using the dendrite/soma label DenMark (magenta) and the presynaptic label synaptotagmin::GFP (Syt::GFP, green). Substacks of the regions of interest for each GFC are shown for DenMark (column 1) and Syt::GFP (column 2), with above and below paired comparisons (image column 3). Arrows indicate the position of the IB. GFC schematic representations are shown (center column), with regions of interest outlined in black boxes. ***A***, GFC1 (78A06-Gal4) processes are labeled by presynaptic Syt::GFP in both TG1 (top) and TG2 (bottom) segments, while the IB is labeled by postsynaptic DenMark. ***B***, GFC2 (73C07-Gal4) processes in TG2 (column 1) are colabeled by both DenMark (column 1) and the Syt::GFP marker (column 2). The IB is labeled by presynaptic Syt::GFP, but also has the DenMark signal (column 4). ***C***, GFC3 (24H07-Gal4) has punctate Syt::GFP within the finger-like processes in TG3 (column 2). The IB is labeled by DenMark, with no Syt::GFP marker (column 4). GFC3 processes along the GFI axonal bend also express the DenMark label (arrowheads).

To investigate GFC postsynaptic domains, we used the UAS-*DenMark* dendrite reporter, composed of the exogenous mouse intercellular adhesion molecule-5 dendritic protein fused to RFP (Nicolai et al., 2010). For presynaptic labeling, we used the UAS-*synaptotagmin::GFP* (Syt::GFP) reporter, composed of the Syt1 integral synaptic vesicle protein fused to GFP (Zhang et al., 2002). In GFC1, the DenMark signal is absent from the finger-like projections at the process termini (Fig. 5*A*, column 1), and Syt::GFP is strongly present in a punctate array, indicating that these processes are presynaptic sites (Fig. 5*A*, column 2). In contrast, DenMark strongly labels GFC1 within the IB (Fig. 5*A*, arrow), indicating that this site is postsynaptic to the GFI (Fig. 5*A*, image column 3, top). The Syt::GFP signal is absent (Fig. 5*A*, image column 3, bottom), suggesting that the IB site is solely for input. Together, these data indicate that GFC1 neurons receive presynaptic input into their dendrites at the IB and then project their contralateral axons for synaptic output into the leg neuropil (Namiki et al., 2018).

In contrast, GFC2 looped processes are strongly labeled by DenMark, including contacts at the GFI axon bend (Fig. 5*B*, column 1), with strongly colocalizing Syt::GFP (Fig. 5*B*, column 2). Only the dorsolaterally projecting processes in the wing neuropil display Syt::GFP without DenMark present. Similarly within the IB, DenMark and Syt::GFP again colocalize, although DenMark is at a low level (Fig. 5*B*, image column 3). Thus, GFC2 neurons appear to have many colocalized presynaptic and postsynaptic domains. Note that it is not possible to tell where in the loop GFC2 processes double back, and the presynaptic and postsynaptic compartments may be in separate, adjacent processes (Fig. 5*B*). Based on our ShakB findings (Fig. 4*B*), it is likely that GFI and GFC2 directly synapse, but both appear presynaptic at the IB, and they may also share postsynaptic targets that mediate GFI–GFC2 coupling. Another possibility is that GFI–GFC2 dye transfer does not occur at the IB, but instead they couple indirectly via an intermediary neuron. This could explain why the GFC2 is relatively poorly labeled by NB dye injection into the GFI, compared with other GFCs.

GFC3 has preynaptic and postsynaptic domains similar to GFC1 (Fig. 5*C*). The GFC3 long finger-like process projections in TG3 have a very weak DenMark signal (Fig. 5*C*, column 1) and very clear Syt::GFP punctae (Fig. 5*C*, column 2). Therefore, these sites are presumably presynaptic in leg neuropil (Namiki et al., 2018). At the IB, GFC3 strongly expresses DenMark (Fig. 5*C*, image column 3), which is thus postsynaptic. However, Denmark expression expands beyond the IB to include GFC3 branches that parallel the GFI axon bend and descending processes (Fig. 5*C*, arrowheads). Syt:GFP is undetectable at all of these GFC3 sites, indicating that they are solely postsynaptic (Fig. 5*C*, image column 3). Surprisingly, DenMark/Syt::GFP expression is lethal with the 42A06-Gal4 driver, and we were therefore unable to evaluate GFC4 presynaptic and postsynaptic domains. Based on similarities to GFC3, we predict that GFC4 has postsynaptic sites at the IB and presynaptic sites in the TG1 leg neuropil. Overall, DenMark and Syt::GFP clearly distinguish presynaptic and postsynaptic regions of all GFC neurons, except GFC2. As the GFCs are so intimately interconnected with the GFI, we next tested whether these coupled neurons play a role in GF circuit development or maintenance.

### GFC requirements for the development of GF circuit architecture

**Figure 6.**
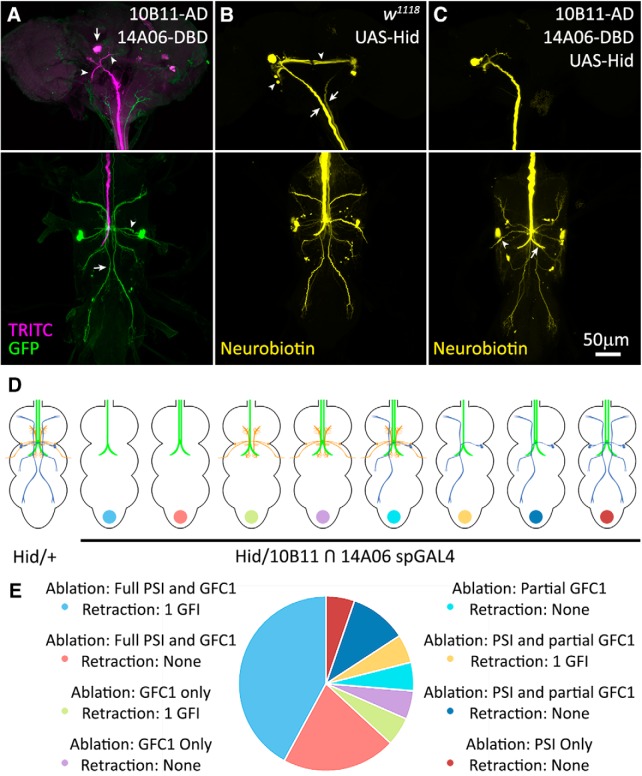
GFC neurons support GF circuit architectural development. ***A***, The GFI labeled by iontophoretically injected TRITC (magenta) reveals the soma (arrow) and dendritic branches (arrowheads) in the brain (top), and descending axon in thoracic ganglion (bottom). Split Gal4 (spGal4) 10B11-AD ∩ 14A06-DBD drives UAS-*mCD8::GFP* (green) in GFC1 (bottom, arrow) and PSI (bottom, arrowhead). ***B***, Iontophoretic NB injection into the GFI (yellow) in the UAS-*hid*/+ control reveals the GFI (arrows) interconnected by the GCI (arrowheads) in the brain (top) and normal dye coupling in the thoracic ganglion (bottom). ***C***, Driving UAS-*hid* with spGal4 10B11-AD ∩ 14A06-DBD results in the loss of GFC1 with occasional PSI survival (arrowhead). When GFC1 is ablated, the GCI labeling is often lost (top), one of the GFI axons is typically absent, and the remaining GFI axon always extends a compensatory contralateral axon projection (arrow). All NB injections were performed on males. ***D***, Schematic representations of GF circuit outcomes with UAS-*hid*/+ controls and spGal4 10B11-AD ∩ 14A06-DBD-driven UAS-*hid* cell ablation. Not pictured are instances where neither GFC1 nor PSI are ablated, and instances where both GFIs are absent. ***E***, Frequency of each GF circuit outcome with the targeted spGal4 10B11-AD ∩ 14A06-DBD-driven UAS-*hid* cell ablation. The pie chart color is coded to dots at the bottom of schematics in ***D***. The sample size for UAS-*hid*/+ genetic controls is 21 animals, and for the spGal4 cell ablation it is 20 animals.

We used Gal4-targeted expression of the Hid protein to drive apoptosis in GFC neurons, in an attempt to eliminate each GFC neuron and study the effects on the GF circuit architecture (Zhou et al., 1997; Muthukumar et al., 2014). Unfortunately, all of the GFC drivers used above (Fig. 2) are lethal in combination with UAS*-hid*. We repeated the study using split-Gal4 (spGal4) lines 10B11-AD ∩ 14A06-DBD (Luan et al., 2006; Pfeiffer et al., 2010; Dionne et al., 2018) to eliminate the apoptosis of off-target cells. These spGal4 lines were identified using the MIP search tool and were selected for their strong expression in GFC1 with minimal overlap in nonspecific neurons. This spGal4 combination expresses strongly in GFC1, but also in PSI, as seen when crossed with UAS-*mCD8::GFP* (Fig. 6*A*, green) with injected TRITC (Fig. 6*A*, magenta) to label the GFI. In the brain (Fig. 6*A*, top), only TRITC dye is present in the GFI, where the GFI cell bodies (Fig. 6*A*, arrow) and their dendrites (arrowheads) reside. Importantly, no mCD8::GFP is present in the GFI (Fig. 6*A*, green). Similarly, the giant commissural interneuron (GCI), which interconnects the GFIs, displays no mCD8::GFP. In the TG, GFC1 (Fig. 6*A*, arrow) and PSI (Fig. 6*A*, arrowhead) express mCD8::GFP (Fig. 6*A*, bottom).

NB dye injection into GFI in a UAS-*hid*/+ control animal shows both GFIs labeled in the brain (Fig. 6*B*, arrows). The GCI (Fig. 6*B*, arrowheads) interconnecting the GFI cell bodies (Allen et al., 1998) is also dye labeled. In the TG, the intact dye-coupled GF circuit is present in all UAS*-hid*/+ control animals (Fig. 6*B*, bottom). When the spGal4 driver is crossed to UAS*-hid* and the GFI injected with NB, GFC1 is ablated in 18 of 20 animals (90%); fully in 14 of 20 animals, partially in 4 of 20 animals (Fig. 6*C*). Partial ablations are defined as several, but not all, neurons within GFC1 clusters being killed. PSI is eliminated in 16 of 20 animals (80%). Two animals had no visible CC axons and could not be injected for analysis. The ablation of coupled cells causes stronger dye labeling in the persisting neurons, as expected due to the reduced volume of the GF circuit. As a consequence, the standard 2 min NB dye injection can cause lysis of the GF circuit, and therefore injection times were reduced to ≤30 s for these ablation experiments. This finding is similar to previous reports when GFI dye coupling is eliminated through lack of interconnecting gap junctions (Kennedy and Broadie, 2017).

When testing the GF circuit for connectivity changes, we find GFC1/PSI ablation causes a striking impact on GFI development (Fig. 6*C*). All control animals (UAS-*hid*/+, *n* = 21) display a completely normal dye-coupled GF circuit without detectable defects (Fig. 6*B*). With targeted UAS-*hid* ablation (spGal4 10B11-AD ∩ 14A06-DBD>UAS-*hid*, *n* = 20 animals), in 9 of 14 animals (∼65%) with complete GFC1 ablation (including one case with the PSI present; Fig. 6*C*, arrowhead), one of the GFI neurons is completely absent (Fig. 6*D*,*E*). In partial GFC1 ablation cases, only one of four animals (25%) lost a GFI. When a GFI is lost, there is no visible dye within the neuron, including the soma and the axon (Fig. 6*C*), and we detect only one axon traveling through the CC by light microscopy. The remaining GFI always extends a compensating axon to the contralateral side (10 of 10 animals; 100%) and forms a normal terminal axon bend (Fig. 6*C*, arrow).

Targeted UAS-*hid* expression is restricted to GFC1 and PS1, with no evidence of either GFI or GCI expression. A full summary of the experimental results is compared between UAS-*hid*/+ controls (*n* = 21) and the spGal4 10B11-AD ∩ 14A06-DBD>UAS-*hid* targeted ablation (*n* = 20; Fig. 6*D*,*E*). Interestingly, in an animal with a fully intact GFC1 and only PSI ablation, both GFIs are present. In an animal with neither PSI nor GFC1 ablated, both GFIs are present (Fig. 6*D*,*E*). PSI ablation alone does not appear to be responsible for GFI loss, as GFI loss occurs when GFC1 alone is missing, but not when PSI alone is missing. We therefore conclude that GFC1 helps to maintain GFI during GF circuit development. Another interesting ablation result is the loss of GFI dye coupling to GCI in 5 of 10 animals (50%) where a GFI is lost (Fig. 6*C*). Surprisingly, this loss of GCI also occurs in two animals where both GFIs are present; one with only GFC1 ablated, and the other with only PSI ablated. These results suggest the GFC neurons, alongside the classic GF circuit neurons, play an important role in neural circuit development.

## Discussion

We describe here newly discovered neurons in the classic *Drosophila* GF neural circuit (Power, 1948; Sun and Wyman, 1997; Jacobs et al., 2000; Allen et al., 2006) by characterizing four GFC neuron clusters. We identify specific transgenic drivers to both label and manipulate GFC1–4, and map neuronal architecture and polarity. We show that these neurons couple to the GFI via ShakB *N* + 16 innexin (Phelan et al., 2008) primarily at the central IB (Allen et al., 1998), but also at the downstream axonal bend. Alongside the already well established benefits of this circuit, including the large cell size, genetic malleability, and accessible functional/behavioral readouts (Power, 1948; Tanouye and Wyman, 1980; Phelan et al., 1996; Trimarchi et al., 1999), this expanded set of coupled neurons can aid future experiments in neurodevelopment, such as the study of axonal selection between multiple dendritic partners. This circuit map could be further refined using advanced tools, such as MultiColor FlpOut (Nern et al., 2015), as was recently accomplished for *Drosophila* brain descending neurons (Namiki et al., 2018).

This detailed circuit map is most useful for genetic analyses of electrical synapse partner connectivity between individually defined neurons. The GFCs identified in this study are composed of two to seven bilaterally symmetrical neurons clustered on each side of the TG segments. Similar clusters of repeated neurons with apparent connectivity redundancy have been recently identified in *Drosophila* brain descending neurons, where it is also unclear why neurons have such tightly overlapping projection patterns (Namiki et al., 2018). We have insufficient resolution to determine whether the GFC neurons truly are duplicates, or whether they have distinct, proximally adjacent synaptic targets, like the closely overlapping Kenyon cells of the adult brain mushroom body (Crittenden et al., 1998). It has been proposed that neuron duplication may allow for a sliding scale of response within a circuit, whereby more neurons are activated to increase the strength of the response (Namiki et al., 2018). Alternatively, if the neurons contact similar proximal synaptic targets, their role may be to provide ultrafine control of muscle movement in the GF circuit escape response (Namiki et al., 2018).

Complex leg and wing movements are thought to be controlled by extensive TG neural circuits, which are activated by a small number of descending neurons, including the GFI dedicated to rapid escape behavior (Cardona et al., 2009; Hsu and Bhandawat, 2016; Cande et al., 2018; Namiki et al., 2018). The roles of GFC neurons uncovered here have yet to be elucidated, although their electrical coupling to the GFI strongly suggests a close relationship to behaviors promoting or otherwise facilitating the rapid escape jump-and-flight response. Our preliminary attempts to optogenetically activate the GFC neurons through blue-light stimulation of Gal4-targeted ChR2-H134R (Nagel et al., 2005) or ChOP-XXL (Dawydow et al., 2014) channels did not produce behaviors. We suspect the stimulation paradigm was not strong enough, that appropriate sensory costimulation conditions may not have been provided (von Reyn et al., 2014), that behavioral scoring methods were not sensitive enough to detect subtle motor output changes (Cande et al., 2018), or that these neurons modulate internal processes not directly manifest in rapid escape behavior (Joseph et al., 2017).

Based on the very recently proposed ventral nerve cord regional map (Namiki et al., 2018), the most likely targets of the four GFCs identified here are the TG1–3 leg neuropils. GFC2 also appears to target the TG2 wing neuropil. Both leg and wing outputs are integral to the GF circuit escape response (von Reyn et al., 2014). GFC1 targets all three TG leg neuropil segments; GFC2 targets TG2; and GFC3 and GFC4 target TG3 and TG1, respectively. This extensive leg neuropil connectivity may regulate tension in the front and hind legs, allowing the central legs to execute a more effective escape jump (Trimarchi and Schneiderman, 1993; von Reyn et al., 2014; Namiki et al., 2018). In support of this hypothesis, our work indicates that GFCs 1–3 are all directly gap junction coupled to the descending GFI, receiving input primarily at the IB, and thus share in the rapid conduction speed of the GF circuit (Phelan et al., 2008). Further, GFC3 neurons extend postsynaptic processes that parallel the PSI processes, indicating GFC3 may collect input from multiple neurons in the GF circuit.

Like the PSI, all four GFCs appear to synapse on their downstream targets via only chemical synapses, based on Syt::GFP synaptic vesicle marker and lack of ShakB electrical synapse labeling at GFC termini (Allen et al., 2006). It might appear possible that another innexin could mediate these GFC connections (Stebbings et al., 2002; Phelan, 2005); however, the complete absence of dye coupling to neurons downstream of GFCs indicates electrical synapses are absent. In contrast to the other GFCs, GFC2 appears both presynaptic and postsynaptic at the IB connectivity hub, suggesting that it may share postsynaptic partners with GFI, potentially including GFC1, 3, and 4 and/or PSI. Given this circuit connectivity, GFC2 may trigger the rapid escape jump reflex independently of the GFI, in a parallel circuit output long speculated to exist, but not previously identified (Trimarchi and Schneiderman, 1995; Fotowat et al., 2009). Indeed, GFC2 extends presynaptic processes into the tergotrochanteral motoneuron dendritic field, thus mimicking GFI connectivity (King and Wyman, 1980).

DenMark and Syt::GFP reporters are extremely useful in defining neuron polarity (Zhang et al., 2002; Nicolai et al., 2010; Bidaye et al., 2014; Frank et al., 2015), but they have limitations that can make interpretation difficult. Both reporters preferentially mark appropriate synaptic regions, but can mislocalize due to transgenic overexpression (Chen et al., 2014; Kanca et al., 2017). A likely example here is dim DenMark signal near bright Syt::GFP punctae (Fig. 5*C*). The DenMark signal-to-noise ratio is much worse than the IB labeling, while the Syt::GFP signal-to-noise ratio is much stronger; hence, our conclusion that this region is presynaptic. A more problematic example may be the DenMark/Syt::GFP overlap in GFC2 (Fig. 5*B*). This labeling likely shows adjacent presynaptic and postsynaptic processes, which we cannot distinguish; although shared compartments have been reported in mushroom body Kenyon cells (Christiansen et al., 2011; Zheng et al., 2018). It is also worth noting that the 73C07-Gal4 line for GFC2 is the strongest driver used and may cause DenMark or Syt::GFP mislocalization via transgenic overexpression (Chen et al., 2014; Kanca et al., 2017). The 42A06-Gal4 driver for GFC3/4 is lethal with UAS-*DenMark*, *syt::GFP*, showing that these markers can also have detrimental effects.

Our targeted ablation studies indicate a role for GFCs in GF circuit development, and demonstrate the ability of the circuit to compensate for the loss of a GFI, much like ocular dominance columns in the classic work by Hubel and Wiesel (1970) and Hubel et al. (1977). PSI ablation does not appear to be responsible for the GFI loss, based on the fact that GFIs are present when PSI alone is ablated, and GFIs are lost only when GFC1 is ablated. Another impact of ablation is lost GCI coupling when a GFI, GFC1, or PSI is removed. As GCI coupling loss occurs both when GFC1 alone is lost and when PSI alone is lost, it appears that complete GF circuit formation depends on feedback from multiple circuit members (Kandler and Katz, 1995; Hanganu et al., 2009; Maher et al., 2009; Belousov and Fontes, 2013). This finding suggests neurons not directly coupled can feedback through an intermediary circuit neuron; an intriguing but poorly studied hypothesis (Kandler and Katz, 1995; Belousov and Fontes, 2013). We note that the TTMn only occasionally dye couples with GFI, suggesting gap junction transitions between open and closed states could also contribute.

Previous studies have shown ablation of the GFI using neurotoxins, such as ricin (Smith et al., 1996), and have even found that single GFIs are lost at very low frequency in wild-type animals (Allen et al., 1998). In the latter case, the authors also found midline crossing of a compensatory contralateral process from the enduring GFI, as in our work. We hypothesize that the GFI loss reported here results from lost GFI stabilization by GFC1 due to the loss of trophic/synaptic signaling or physical contact (Gorin and Johnson, 1979; Pearson and Stoffler, 1992; Antonini and Stryker, 1993; Crowley et al., 1994; Uesaka, 2005; Gibson and Ma, 2011). Other GFI postsynaptic targets (PSI, TTMn, GFC2–4) presumably also participate in GFI stabilization, although Gal4 drivers tested thus far for these neurons have proved lethal in combination with UAS-*hid* (Zhou et al., 1997; Muthukumar et al., 2014). These animals die early in development, showing the need for spGal4 lines capable of avoiding off-target cells. Pursuing this phenotype with more specific drivers and screening approaches could elucidate molecular mechanisms that these neurons use to stabilize synaptic partners (Cohen-Cory, 2002).

Other methods shown to cause GFI axonal retraction and neuronal loss include blocking membrane endocytosis (e.g., using dominant-negative *shibire*/Dynamin) and the overexpression of select transmembrane receptors, such as semaphorin-1A (Godenschwege et al., 2002; Murphey, 2003; Godenschwege and Murphey, 2009). However, in these cases, GFI axon retraction is typically only to the IB, rather than beyond the CC, or causing complete cell loss. The molecular pathways responsible for these phenotypes may be shared with the axon retraction caused by the loss of synaptic partners, with Highwire/MYCBP2, Wallenda/DLK, and Basket/JNK as prime candidates (Ghosh et al., 2011; Borgen et al., 2017). While gap junctions play extensive roles in neuronal development (Elias and Kriegstein, 2008; Belousov and Fontes, 2013; Baker and Macagno, 2017), it is unlikely that GFI loss results from the loss of electrical coupling only, as the total removal of gap junctions from the GFI does not cause axon retraction or neuronal cell death (Blagburn et al., 1999).

The GFI axon split across the midline in response to the absence of its partner is reminiscent of sensory neuron plasticity following input deprivation (Poirier et al., 2006; Collignon et al., 2009; Rabinowitch et al., 2016) and motor circuit development changes in response to lost motor neurons (Modney and Muller, 1994; Büschges et al., 2000). This corrective rewiring could stem from either normal pathfinding and synaptogenesis or new repair pathways activated in response to unpartnered neurons. The axon split duplication with a GFI loss is different from the recent report on failed GFI pruning (Borgen et al., 2017), as the new GFI axon path is always a perfect mirror image of the normal axon bend, rather than an untrimmed posteriorly branched axon outgrowth. This new circuit rewiring model could be used in *Drosophila* genetic screens of GF circuit development (Mohr, 2014; Bassett et al., 2015; Heigwer et al., 2018) to help answer a number of important questions. Such work will be greatly aided by single-cell transgenic manipulation of presynaptic and postsynaptic neurons in the GF circuit.

In conclusion, we hope that the increase in manipulatable GFI-coupled neurons reported here will further enhance this genetic model circuit. The GF circuit is ideally suited to query a wide range of important neurodevelopmental questions, including mechanisms of pathfinding, target recognition, synaptogenesis, and stabilization during neural circuit assembly and maintenance. Although the GF circuit is rightly considered one of the most straightforward and accessible *Drosophila* circuits, the higher degree of connectivity revealed in this study indicates a greater complexity, which is amenable to answering more in-depth questions. The large number of inputs onto, and outputs from, this model circuit provides further evidence that even the most basic circuits are deeply interconnected with the rest of the brain circuitry. As the benefits of single-cell resolution studies cannot be overstated, we hope that this enlarged GF circuit model, and the transgenic tools characterized here, will help to form part of the underpinning for future work on neural circuit dynamics.

## Synthesis

Reviewing Editor: Muriel Thoby-Brisson, CNRS UMR 5287 Université Bordeaux Decisions are customarily a result of the Reviewing Editor and the peer reviewers coming together and discussing their recommendations until a consensus is reached. When revisions are invited, a fact-based synthesis statement explaining their decision and outlining what is needed to prepare a revision will be listed below. The following reviewer(s) agreed to reveal their identity: Till Bockermuehl.

Dear Authors,

Please find below the comments of the two reviewers who evaluated your submission. We all agree on the potential interest of the study and we have only minor comments. Great paper.

Reviewer 1:

The manuscript entitled “Newly Identified Electrically Coupled Neurons Support Development of the Drosophila Giant Fiber Model Circuit” (eN-NWR-0346-18) seeks to expand the framework by which we understand the function of the Giant Fiber Escape pathway in Drosophila. This is a well-studied circuit and so taking a systematic approach to expand the neural elements that comprise this circuit is a valuable addition to the field. This paper is technically sound, well written, systematic and provides an excellent degree of detail in it's methods making replication easy for anyone familiar with using Drosophila. I have only minor comments and any comments along the lines of “you could try this” are not meant for this study, but potentially for future work.

Minor comments

-Figure 3. Each channel for a specific GFC cluster should be pseudocolored to match the color scheme used for the cartoon schematics. Right now, they are all green. This will make it easier for the reader to connect the individual Gal4 lines ID'd with the cartoon representation on the right.

-Figure 4. Please recolor the red channel for Denmark with magenta to accommodate people with red/green color blindness.

-I was unclear as to the logic of the order in which the data for figures 3-5 are presented, and it may be easier to follow if 5 follows 3 rather than having 4 interleaving. The purpose of figure 3 is to infer sites of connectivity between GFIs and GFC clusters based on physical overlap. Figure 5 shows overlap between GFIs, GFCs and ShakerB labeling, which shows where presumed sites of electrical coupling occur. Figures 3 and 5 are therefore complementary. Figure 4 on the other hand establishes input and output domains for individual neurons based on chemical signaling. Thus presenting 3 and 5 together may make more sense than with figure 4 in between, as the connectivity demonstrated in 1-3 and 5 is based on electrical coupling, rather than chemical signaling.

-Lines 345-7 should be changed to “Flies in which GFC1-4 neurons are labeled with UAS-mCD8::GFP (Fig. 5, column 1, green) were GFI-injected with TRITC (column 2, magenta) and co-labeled with ShakB antibody (column 3, cyan).”

-MCFO is an excellent idea to pursue for future more detailed analyses of the role of the different GFCs as would using the heat-activated diptheria (DT-A) to kill GFC clusters in adults and then look at the behavioral consequences for looming escape responses. The GFC neurons may play specific roles in dictating the direction or type of take-off elicited. If this were the case optogenetic activation of these GFCs wouldn't have elicited an escape.

Reviewer 2:

In this study, the authors describe several new groups of thoracic neurons that are part of the Giant Fiber circuit in Drosophila. They use several straight-forward techniques (dye injections, confocal fluorescence imaging, and GAL4/UAS-based approaches) to characterize these newly found neuron groups with regard to their morphology, their putative connections to the previously described parts of the circuit, and to investigate developmental effects of targeted ablation.

Having no detailed knowledge about the investigated circuit, I found the paper very easy to read and follow. The reasoning behind the used methods and their interpretation was very clear and the authors did a good job of explaining their findings. In addition, I felt that the characterization was thorough and lays a good groundwork for follow-up studies. For me personally, it will be very interesting to see what the function of these newly described neurons is in the context of escape.

I have only minor comments.

- The language is sometimes overly flowery or powerful. Examples for this are “storied history” (line 2), “epicenter” (line 71), “GFI portal” (line 90), “we set forth” (line 187), “dramatic increase” (line 572), and “formidable genetic model circuit” (line 573). It is clear that the authors are excited about the circuit (and that is very commendable), but for my taste the phrasing is a bit too much; I recommend finding more neutral expressions.

- At various places the authors state that they “added” neurons to the circuit or “expanded” it. While I know what the authors want to say I recommend using more appropriate terms like “found” or “describe”.

- The first paragraph of the introduction (specifically lines 38 to 47) is very generic and has no particular connection to the specific topic of the study. While everything in it is certainly true it does not add anything meaningful in introducing the topic, in my opinion; I would recommend omitting it, or, if you absolutely want to keep the references, work the various general aspects referred to in it into appropriate places in the methods section.

- You found several GAL4 driver lines for GFC1-4; these are used for the subsequent analysis. Table 1 however, lists a completely different set of driver lines, not used in the analysis and only referred to in line 200. The table legend refers to them only as “GAL4 driver lines for the GI circuit”. I suspect that the lines in the table are only referenced as further lines that might be interesting to other researchers, but during the first read I was very confused that these are not the ones you used for analysis. It should be made clearer in the Results (around line 200), that these are just additional ones and are mentioned for completeness. The same goes for Table 1.

- It would be helpful to briefly elaborate on how you found the AD and DBD hemidrivers you used for sparse expression. Did you also use the MIP tool or a different approach? As it is, these driver lines appear out of nowhere.

- In lines 469 to 471 you refer to attempts to activate GFC neurons. This seems to be very preliminary data (since experiments with regards to that are not described in the Methods or Results), so this should be more clearly highlighted as such. As it stands, I was very surprised to read that in the Discussion, since it is not mentioned anywhere else in the manuscript.

- There is a lot of unneeded redundancy in the Figure legends. You already have a very extensive description of, for instance, GFC architecture (lines 215 to 267) in the Results section. Much of it is repeated in the legend to Figure 2. This can be shortened quite a bit. The same is true to some extent for the other Figures.

- Figure 1 (and others): The VNC should not be filled in gray. It does not add anything to the description of the relative arrangement of the different neuron locations and projection; at the same time it reduces contrast. Instead, only use an outline of the VNCs.

## Author Response

We submit here our revised eN-NWR-0346-18 study within the specified revision period. Below, we provide point-by-point responses to the reviewer comments. We appreciate the reviewers' efforts to make our manuscript as clear and concise as possible. We have carefully adhered to the Journal guidelines, so we believe the attached manuscript meets all requirements for publication. Please let us know if anything further is needed.

Reviewer 1

Reviewer 1 writes “This paper is technically sound, well written, systematic and provides an excellent degree of detail in its methods making replication easy for anyone familiar with using Drosophila. I have only minor comments and any comments along the lines of ”you could try this“ are not meant for this study, but potentially for future work.”

Comments:

1. Figure 3: Each channel for a specific GFC cluster should be pseudocolored to match the color scheme used for the cartoon schematics.

As suggested, we made these changes, but the new color was confusing with respect to the fluorophore/technique used, the merged images were more difficult to interpret, and we lost color contrast with neurobiotin for GFC4. Therefore, we have maintained the color scheme for each fluorophore/technique. Note: We trust comment refers to figure 2.

2. Figure 4: Please recolor the red channel for Denmark with magenta to accommodate people with red/green color blindness.

As suggested, we have changed red to magenta throughout this figure.

3. Presenting figures 3 and 5 together may make more sense than with figure 4 in between, as the connectivity demonstrated in 1-3 and 5 is based on electrical coupling.

As suggested, we changed the figure order to place figure 5 between figures 3 and 4. We agree this makes more sense in presenting the data and have updated the text.

4. Lines 345-7 should be changed to “Flies in which GFC1-4 neurons are labeled with UAS-mCD8::GFP (Fig. 5, column 1, green) were GFI-injected with TRITC (column 2, magenta) and co-labeled with ShakB antibody (column 3, cyan).”

As suggested, we have made this change.

Reviewer 2

Reviewer 2 writes “...I found the paper very easy to read and follow. The reasoning behind the used methods and their interpretation was very clear and the authors did a good job of explaining their findings. In addition, I felt the characterization was thorough and lays a good groundwork for follow-up studies...I have only minor comments.”

Comments:

1. The language is sometimes overly flowery or powerful. It is clear that the authors are excited about the circuit (and that is very commendable), but for my taste the phrasing is a bit too much; I recommend finding more neutral expressions.

As suggested, we have revised our writing to more neutral expressions. This includes the instances suggested by the reviewer and other instances throughout the paper.

2. At various places the authors state they “added” neurons to the circuit or “expanded” it. While I know what the authors mean, I recommend using “found” or “describe”.

As suggested, these edits have been made throughout the paper.

3. I recommend omitting the first paragraph of the introduction, as it has no particular connection to the specific topic of the study, while everything in it is certainly true.

As suggested, we have removed the first paragraph of the introduction.

4. Table 1: It should be made clearer in the Results (around line 200), the purpose of these Gal4 lines.

As suggested, we have clarified the source and purpose of these Gal4 lines.

5. It would be helpful to briefly elaborate on how you found the AD and DBD hemidrivers you used for sparse expression. Did you also use the MIP tool or a different approach?

As suggested, we have updated the text to clarify the origin of these lines, which were indeed found using the MIP tool.

6. In lines 469 to 471 you refer to attempts to activate GFC neurons. This seems to be very preliminary data (since experiments with regards to that are not described in the Methods or Results), so this should be more clearly highlighted as such.

As suggested, we have revised the paper to clarify these are very preliminary results. Appropriate positive and negative controls worked, but were unable to get any notable findings. We feel the Discussion is the most appropriate place to indicate attempts had been made, so as not to suggest these experiments have been executed to completion. Our hope is to encourage further research with recently developed, more advanced behavioral assay methods (e.g. Cande, et al. 2018).

7. There is a lot of unneeded redundancy in the figure legends, especially for figure 2. This can be shortened quite a bit. The same is true to some extent for the other figures.

As suggested, the figure legends have been revised to reduce redundancy and make them both shorter and crisper.

8. Figure 1 (and others): The VNC should not be filled in gray. It does not add anything to description of the relative arrangement of different neuron locations and projections; at the same time it reduces contrast. Instead, only use an outline of the VNCs.

As suggested, we have revised the VNC schematics to a simple, white background in all figures, and agree the presentation provides better contrast.
